# An Edge–Cloud Collaborative ECG-Assisted Diagnostic System Leveraging Cross-Lead Knowledge Distillation and Large Language Models

**DOI:** 10.3390/s26123753

**Published:** 2026-06-12

**Authors:** Haohan Su, Shuai Wang, Hongxiao Wang, Keni Qiu

**Affiliations:** Information Engineering College, Capital Normal University, Beijing 100048, China

**Keywords:** electrocardiogram (ECG), knowledge distillation, large language model, edge–cloud collaboration, wearable device, LoRA fine-tuning

## Abstract

**Highlights:**

**What are the main findings?**

- Cross-lead distillation compresses a 12-lead teacher into a 6417-parameter single-lead student, retaining 92.8% Macro-F1 and 94.7% AUC-ROC.
- LoRA fine-tuning of Qwen3-8B improves report structural completeness and end-to-end consistency over the base model.

**What is the implication of the main finding?**

- The InceptionTime–Transformer hybrid teacher captures both multi-scale local morphology and global temporal dependencies, yielding more informative soft labels for cross-lead distillation.
- The edge–cloud architecture provides a reference design for combining on-device screening with LLM-based interpretable analysis.

**Abstract:**

Cardiovascular diseases impose a substantial global health burden and often require timely detection, creating strong demand for real-time electrocardiogram (ECG) monitoring on resource-constrained devices. Although portable single-lead wearable ECG devices are valuable for daily monitoring, their diagnostic performance is limited by spatial information loss and hardware constraints. Moreover, conventional lightweight models lack interpretable analysis beyond coarse classification. This study proposes an edge–cloud collaborative ECG-assisted analysis method combining lightweight ECG model distillation with large language models. At the algorithmic level, a cross-lead distillation framework transfers knowledge from a 12-lead InceptionTime–Transformer teacher to an ultra-lightweight single-lead student via a hybrid loss integrating hard-label, temperature-scaled soft-label, and auxiliary multi-label objectives. At the system level, a three-layer architecture integrates edge-side real-time screening with cloud-side report generation through a LoRA-fine-tuned Qwen3-8B model. Experiments on PTB-XL show that, under 123.7× parameter compression and 12-to-1 lead reduction, the student retains 92.8% of the teacher’s Macro-F1 and 94.7% of its AUC-ROC. After 8-bit integer (INT8) quantization, the TFLite file is 20.8 KB; QEMU-based Cortex-M4 simulation shows approximately 63.0 KB SRAM usage and 11.6 ms latency, suggesting potential on-device deployment under simulated conditions. Validation on physical hardware—including power consumption, BLE latency, and motion artifacts—remains necessary.

## 1. Introduction

Cardiovascular disease remains the leading cause of death worldwide. According to the Global Burden of Disease 2023 study, it claimed approximately 19.2 million lives in 2023, accounting for one in three deaths globally [1,2]. Since many acute cardiovascular events are highly time-sensitive, early detection, timely warning, and rapid intervention are essential for improving survival and clinical outcomes [3,4]. In recent years, with the rapid development of Internet of Things (IoT) technologies and intelligent algorithms, portable wearable ECG devices have been increasingly adopted for daily health monitoring because of their non-invasive nature, low power consumption, and suitability for long-term use [5,6]. However, compared with standard clinical 12-lead ECG systems, existing single-lead wearable devices still face two major challenges in practical applications. First, they suffer from insufficient data dimensionality. A single-lead signal reflects cardiac electrophysiological activity from only one spatial perspective and cannot capture the richer spatial vector information available in multi-lead recordings. As a result, the detection performance of such devices may be limited for certain complex arrhythmias and structural cardiac abnormalities [7,8]. Second, edge-side hardware resources are severely constrained. Microcontrollers deployed in wearable devices are typically limited in computation capability, memory capacity, and energy budget, making it difficult to directly deploy high-accuracy deep learning models with large parameter scales [9]. In addition, most lightweight edge-side classifiers provide only coarse binary outputs, such as “normal” or “abnormal”, and lack the ability to generate more detailed and interpretable health feedback for users [10].

To improve the performance of single-lead wearable ECG analysis, existing studies have mainly focused on developing highly lightweight convolutional neural networks (CNNs) or recurrent neural networks (RNNs) for on-device deployment [11]. Although such approaches can reduce model size, aggressive compression through pruning or quantization may lead to a loss of diagnostic accuracy. Meanwhile, with the rapid progress of large language models (LLMs) in medical natural language processing, increasing attention has been given to their potential use in auxiliary diagnosis and medical report generation [12]. In ECG-assisted analysis, LLMs are often used not as standalone waveform classifiers, but as reasoning and generation modules that operate on extracted signal features, preliminary model outputs, or structured clinical context to support ECG interpretation, risk explanation, and report generation. This makes them useful complements to conventional lightweight classifiers, which often provide only coarse classification labels and limited explanatory feedback. Nevertheless, raw ECG signals can easily exceed the context window of current language models [13], and the computational and memory requirements of current large-scale LLMs generally preclude their direct deployment on resource-constrained edge devices [14]. Therefore, a key challenge is how to achieve accurate and low-latency preliminary screening at the edge while exploiting the stronger reasoning and generation capabilities of cloud-based LLMs to provide clinically meaningful interpretation.

**Figure 1:**
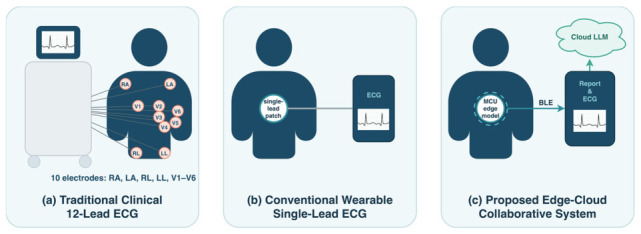
Comparison of ECG diagnostic systems: (**a**) traditional clinical 12-lead ECG, which provides high diagnostic accuracy but has limited portability; (**b**) conventional wearable single-lead ECG, which is portable but offers limited diagnostic depth; and (**c**) the proposed edge–cloud collaborative system, which combines edge-side real-time warning with cloud-side LLM-based deep analysis. In (**a**), the electrode placement labels denote standard ECG lead positions: RA (right arm), LA (left arm), RL (right leg), LL (left leg), and V1–V6 (precordial chest leads).

To address this issue, this paper proposes an edge–cloud integrated ECG-assisted analysis method based on the collaboration of knowledge distillation and large language models, and evaluates its preliminary technical viability through a proof-of-concept prototype and QEMU-based edge simulation. As illustrated in Figure 1, unlike conventional clinical 12-lead ECG systems and existing single-lead wearable devices, the proposed framework adopts a dual-model architecture that combines edge-side preliminary screening with cloud-side deep analysis.

At the edge perception layer, a cross-lead knowledge distillation framework is constructed [15]. Specifically, a 12-lead InceptionTime [16]-Transformer [17] hybrid network is employed as the teacher model. This design combines multi-scale local feature extraction with global temporal dependency modeling, aiming to provide informative soft-label supervision for the single-lead student. An ultra-lightweight student network based on depthwise separable convolution is designed for single-lead input.

To transfer the teacher’s diagnostic knowledge to the student, a hybrid training objective is introduced, integrating hard-label supervision, temperature-scaled soft-label distillation, and auxiliary multi-label distillation. The auxiliary multi-label branch is used only during training and is removed during inference, thereby introducing no additional runtime overhead. Detailed model design is presented in Section 3.

At the cloud analysis layer, a Qwen3-8B [18] large language model fine-tuned with LoRA [19] is deployed to generate structured health analysis reports. The deployed edge-side student model provides a binary abnormality probability, which is combined with real-time physiological indicators, including average heart rate and standard deviation of NN intervals (SDNN), and user background information such as age, gender, and medical history to form the cloud-side structured prompt. The model produces personalized reports whose content varies with individual patient context even when the binary screening labels are identical. The detailed design of this module is described in Section 4. By leveraging chain-of-thought prompting, the cloud-side LLM is designed to generate more structured and contextually detailed auxiliary reports than the coarse binary outputs of conventional lightweight classifiers [20].

The main contributions of this study are summarized as follows:

1. A cross-lead knowledge distillation framework is proposed to transfer diagnostic knowledge from a 12-lead teacher model to an ultra-lightweight single-lead DSConv [21] student model. By exploiting richer spatial information during training, the framework aims to reduce the performance degradation caused by single-lead acquisition. The distilled model shows compatibility with Cortex-M4-class storage and memory constraints in QEMU-based simulation, while real-hardware validation remains to be conducted.
2. An edge–cloud collaborative prototype is developed to connect on-device preliminary screening, physiological feature extraction, and cloud-side report generation into a unified workflow. A LoRA-fine-tuned Qwen3-8B model is used as the cloud analysis engine to generate structured health reports from edge-side screening results and physiological indicators.
3. A deployment-aligned prompt construction and fine-tuning strategy is designed to embed edge-side screening results, physiological indicators, and user background information into structured prompts. This strategy improves the adaptability of the cloud-side LLM to the actual system input format and supports more consistent report generation in the proposed prototype workflow.

The remainder of this paper is organized as follows. Section 2 reviews ECG signal analysis, deep-learning-based ECG diagnosis, knowledge distillation, and medical large language models, and summarizes the limitations of existing work. Section 3 presents the proposed cross-lead knowledge distillation framework. Section 4 describes the edge–cloud collaborative system design and prototype implementation. Section 5 reports the experimental results, ablation studies, efficiency analysis, and cloud-side LLM evaluation. Finally, Section 6 concludes the paper and discusses limitations and future directions.

## 2. Background and Related Works

### 2.1. Fundamentals of ECG Signals and Limitations of Single-Lead Recording

**Figure 2:**
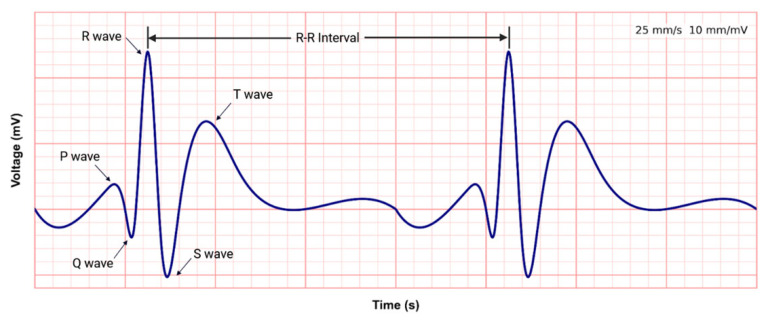
A standard ECG waveform illustrating the P wave, QRS complex, T wave, and the R–R interval used for heart rate and heart rate variability (HRV) calculations.

An ECG is a noninvasive tool for recording the temporal variation in cardiac electrical activity. As illustrated in Figure 2, a standard cardiac cycle consists of the P wave, QRS complex, and T wave, whose morphology, amplitude, and duration reflect the depolarization and repolarization processes of the atria and ventricles [22].

Portable wearable devices typically adopt a single-lead configuration and have been widely used in daily monitoring scenarios [23]. However, such a configuration has inherent limitations. First, a single lead captures cardiac electrical activity from only one projection angle and therefore cannot fully reflect the three-dimensional electrophysiological changes in the heart [7]. Second, single-lead signals are more susceptible to baseline wander, electromyographic (EMG) interference, and motion artifacts during ambulatory monitoring, which reduces the signal-to-noise ratio (SNR) and increases the difficulty of automated analysis [24]. In this study, average heart rate and SDNN-based heart rate variability are further used as lightweight physiological indicators for local alerting and cloud-side prompt construction.

### 2.2. Current Status of Deep Learning-Based Intelligent ECG Analysis

In recent years, deep learning has achieved substantial progress in ECG analysis. Models based on CNNs, residual networks, Transformers, and hybrid architectures have reported performance approaching that of clinical experts on several public datasets [25,26,27].

Despite these advances, most high-performance ECG models rely on 12-lead inputs to exploit rich spatial information. When such models are adapted to single-lead inputs, their classification performance often degrades markedly [28]. This dependence on multi-lead signals limits the direct deployment of existing high-accuracy models on mainstream wearable devices, which are predominantly single-lead. This gap motivates the present study: how to transfer the diagnostic knowledge embedded in multi-lead signals to a single-lead model without increasing edge-side hardware cost.

The choice of teacher architecture directly influences cross-lead knowledge transfer quality. InceptionTime [16] captures ECG morphological patterns at multiple temporal scales via parallel multi-kernel convolutions, but its purely convolutional structure limits long-range inter-beat dependency modeling. Incorporating Transformer-based self-attention [17] complements this limitation by enabling global temporal modeling across the full recording. This study therefore adopts an InceptionTime–Transformer hybrid as the teacher; Section 5.2 empirically confirms its advantage over representative CNN-based and recurrent baselines on the PTB-XL benchmark.

### 2.3. Lightweight Models and Knowledge Distillation for Edge Deployment

Knowledge distillation (KD), originally proposed by Hinton et al. [15], trains a compact student model under the guidance of soft targets produced by a larger teacher model and has become an effective paradigm for mitigating performance degradation in lightweight models. In the ECG domain, several studies have begun to explore KD-based compression strategies. For example, Sepahvand et al. employed a 12-lead teacher model to supervise a lightweight model for arrhythmia classification [29].

However, existing ECG distillation studies still mainly focus on moderate model compression, and even prior 12-lead-to-single-lead distillation methods usually retain substantially larger student models. In addition, few studies jointly consider INT8 quantization and microcontroller-level resource evaluation. Therefore, cross-lead distillation under extreme compression and deployment-oriented constraints remains insufficiently explored.

### 2.4. Applications of Large Language Models in Medical Auxiliary Diagnosis

LLMs have shown considerable potential in the medical domain, with encouraging results in clinical text generation, electronic health record (EHR) information extraction, and complex medical question answering [30,31]. However, applying LLMs directly to continuous physiological signal analysis remains challenging. Recent ECG-oriented studies have therefore explored several indirect or multimodal strategies. Yu et al. [32] proposed a retrieval-augmented zero-shot framework that converts extracted ECG features into textual prompts and retrieves cardiology knowledge to support LLM-based diagnosis. Yang et al. [33] developed ECG-LM, which aligns ECG signal representations with the feature space of a large language model for joint ECG–text understanding. Zhao et al. [34] introduced ECG-Chat, a large ECG-language model that combines ECG waveform–text alignment with report generation and conversational diagnosis. Wan et al. [35] proposed the MEIT framework, which applies multimodal instruction tuning to ECG report generation. These studies demonstrate the potential of LLMs to enhance ECG interpretation with more interpretable and context-aware outputs. However, they mainly focus on offline diagnosis, signal–text alignment, or report generation from curated datasets, whereas the present study emphasizes the structured integration of edge-side screening results and physiological indicators into cloud-side LLM inference within an edge–cloud collaborative workflow. Recent studies have shown that carefully designed prompt engineering can substantially improve LLM performance in medical tasks [36], while the use of strong foundation models for synthetic data generation has also been demonstrated to be an effective data augmentation strategy [37,38].

Nevertheless, as noted above, these approaches have not fully addressed the structured integration of real-time physiological indicators with preliminary outputs from lightweight edge models. How to incorporate edge-side screening results as prior knowledge into the LLM inference context, while aligning the fine-tuning data distribution with the actual deployment input format, remains an open problem.

### 2.5. Summary of Limitations in Existing Work

In summary, three gaps remain insufficiently explored: (i) cross-lead distillation under extreme compression with quantization and MCU-level resource evaluation; (ii) structured integration of edge-side screening results and real-time physiological indicators into cloud-side LLM analysis; and (iii) an end-to-end edge–cloud workflow that connects signal acquisition, preliminary screening, feature extraction, cloud-based interpretation, and user feedback.

## 3. Algorithm: Peripheral Lightweight Model Based on Cross-Lead Knowledge Distillation

### 3.1. Problem Analysis and Overall Design

To address the cross-lead extreme compression problem discussed in Section 2.5, this section presents the proposed cross-lead knowledge distillation framework. The framework consists of three main components: data preprocessing, the teacher–student network architectures, and the hybrid distillation training strategy. Specifically, a 12-lead high-capacity teacher model is first trained to learn multi-label diagnostic knowledge from the full ECG representation. Then, a lightweight single-lead student model is trained under the supervision of both ground-truth labels and teacher-generated soft targets. The objective is to preserve the diagnostic knowledge learned from multi-lead ECG signals while enabling efficient binary abnormality screening on resource-constrained edge devices.

### 3.2. Dataset and Preprocessing

#### 3.2.1. Dataset Description

This study adopts the PTB-XL dataset [39] as the experimental benchmark. PTB-XL is one of the largest publicly available 12-lead ECG datasets, containing 21,799 10 s ECG recordings from 18,869 patients. The high-resolution version provides signals sampled at 500 Hz [39,40]. Each record was annotated by multiple cardiology experts according to the SCP-ECG standard, covering 71 fine-grained labels across three categories: diagnostic, form, and rhythm.

To enable the teacher model to learn clinically meaningful pathological representations and provide richer supervision for distillation, this study uses the diagnostic superclass labeling scheme provided by PTB-XL. All records are mapped into five diagnostic superclasses: NORM, MI, STTC, HYP, and CD, corresponding to normal ECG, myocardial infarction, ST/T-segment changes, hypertrophy, and conduction disturbance, respectively. The labels are represented using multi-hot encoding, as shown in Equation (1):

(1) $$y={\left\{0,1\right\}}^{5}$$

where each element indicates whether the corresponding diagnostic superclass is present. This formulation allows a single ECG record to belong to multiple pathological categories simultaneously [40].

#### 3.2.2. Signal Preprocessing

All high-resolution ECG recordings originally sampled at 500 Hz are resampled to 250 Hz using polynomial interpolation, so that the signal frequency is consistent with the target edge acquisition hardware. For each recording, the first 2500 samples are retained, corresponding to a 10 s ECG segment. Records shorter than this fixed length are zero-padded to ensure a uniform model input size.

When a record is simultaneously labeled as NORM and one or more pathological superclasses, the NORM label is removed and the pathological labels are retained. This operation avoids medically inconsistent labeling, since the presence of a pathological diagnosis should override the normal label. Records that cannot be mapped to any of the five diagnostic superclasses are excluded during preprocessing.

For frequency-domain filtering, a third-order Butterworth band-pass filter [41] is applied with a passband of 0.5–100 Hz. The lower cutoff frequency of 0.5 Hz is used to suppress baseline drift, while the upper cutoff frequency of 100 Hz is higher than the conventional 40 Hz upper bound used in many ECG analysis pipelines. Zero-phase forward–backward filtering is used to avoid phase distortion.

For amplitude normalization, this study only performs channel-wise mean subtraction without standard deviation normalization. This design preserves absolute amplitude information relevant to diagnostic patterns such as hypertrophy and ST-T changes [42,43].

### 3.3. Teacher–Student Network Architecture

#### 3.3.1. Teacher Model: InceptionTime–Transformer Hybrid Architecture

The teacher model adopts a hybrid architecture combining convolutional neural networks and Transformers. The convolutional component captures local ECG morphological features at multiple temporal scales, while the Transformer component models long-range temporal dependencies across the full recording [44]. This complementary design allows the teacher to encode both local and global diagnostic information in its soft labels, which is beneficial for cross-lead distillation under single-lead input constraints. The input of the teacher model is a 12-lead ECG segment with shape (B, 2500, 12), where B denotes the batch size.

**Figure 3:**
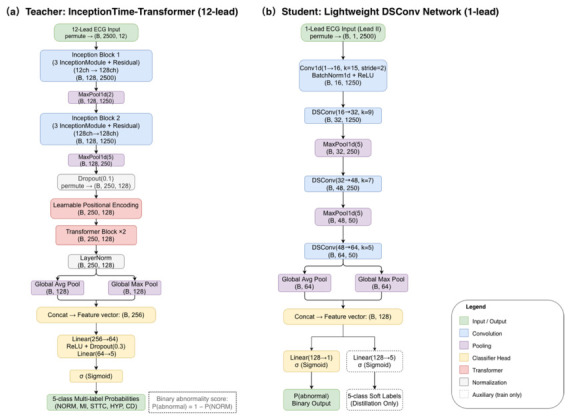
Overall architectures of the Teacher and Student models. (**a**) The Teacher model is a 12-lead InceptionTime–Transformer hybrid network trained with five-class multi-label supervision. (**b**) The Student model is an ultra-lightweight single-lead network based on depthwise separable convolutions (DSConv), with a binary classification head for inference and an auxiliary multi-label head used only during distillation training.

**Figure 4:**
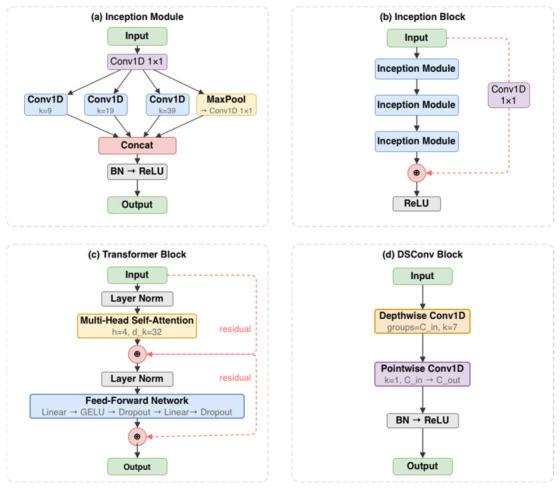
Detailed structures of the key building blocks. (**a**) Inception Module with parallel multi-scale convolution branches using kernel sizes of 9, 19, and 39, together with a max-pooling branch. (**b**) Inception Block consisting of three cascaded Inception Modules and a residual shortcut. (**c**) Pre-Norm Transformer Block with four-head self-attention and a feed-forward network. (**d**) Depthwise Separable Convolution Block, which decomposes standard convolution into depthwise and pointwise operations for parameter efficiency.

In the convolutional feature extraction stage, as shown in Figure 3a, the backbone consists of two cascaded Inception Blocks [16]. Each Inception Block contains three sequential Inception Modules with a residual shortcut connection, as illustrated in Figure 4b.

This multi-scale design enables the model to capture ECG morphological patterns with different temporal durations. The first Inception Block maps the 12 input leads to 128 feature channels and downsamples the temporal length from 2500 to 1250 using MaxPool1d(2). The second Inception Block keeps the channel dimension unchanged and further downsamples the temporal length to 250 using MaxPool1d(5).

In the global dependency modeling stage, the convolutional feature maps are regularized using Dropout(0.1), transposed into a sequence representation with shape (B, 250, 128), and combined with learnable positional embeddings. The resulting sequence is then fed into two Transformer encoder layers. As shown in Figure 4c, each Transformer Block adopts a Pre-Norm architecture [45], consisting of a four-head multi-head self-attention layer and a feed-forward network. The attention head dimension is $d_{k}=32$, the hidden dimension of the feed-forward network is 256, and GELU is used as the activation function. Residual connections are applied around each sublayer.

For classification, the Transformer output is first processed by LayerNorm. Dual global pooling produces a 256-dimensional feature vector. This vector is fed into a two-layer fully connected classifier with dimensions 256 → 64 → 5. The intermediate layer uses ReLU activation and Dropout(0.3), and the final layer outputs five logits. The five-class multi-label probabilities are obtained using the sigmoid function. That is, the hybrid teacher network outputs probabilities for five PTB-XL diagnostic superclasses (NORM, MI, STTC, HYP, and CD), which serve as soft-label supervision during distillation; the NORM probability is further converted into a binary abnormality score via Equation (2) to guide the student’s normal–abnormal screening task.

For binary abnormality estimation, the teacher output is converted based on the NORM class probability. Specifically, the abnormal probability is calculated as shown in Equation (2):

(2) P(abnormal)=1−P(NORM)

#### 3.3.2. Student Model: Lightweight DSConv Network

The student model is designed for lightweight edge deployment and uses only the single-lead Lead II ECG signal as input, with shape (B, 2500, 1). Lead II is selected because it is approximately aligned with the main electrical axis of the heart and usually provides clear P-wave, QRS-complex, and T-wave morphology, making it widely used in clinical rhythm analysis [46]. Accordingly, the current model assumes standard Lead II input; applying it directly to other standard leads or non-standard leads from consumer wearables without lead-specific retraining may lead to degraded or inconsistent performance, as indicated by the lead ablation in Section 5.5.2.

As shown in Figure 3b and Figure 4d, the feature extraction backbone of the student model consists of one standard one-dimensional convolutional layer followed by three depthwise separable convolution modules. DSConv decomposes standard convolution into depthwise and pointwise convolutions [21], thereby reducing the number of parameters and computational cost.

**Figure 5:**
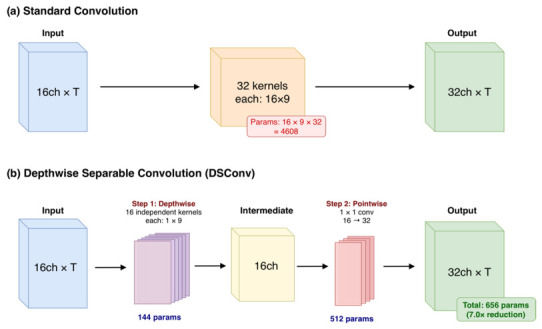
Comparison of computational cost between standard convolution and DSConv, illustrating the parameter reduction achieved by decomposing spatial and channel-wise operations.

As shown in Figure 5, DSConv substantially reduces the parameter count and computational complexity compared with standard convolution [47]. The input signal is first processed by Conv1d(1 → 16, k = 15, stride = 2), batch normalization, and ReLU activation, reducing the temporal length from 2500 to 1250. Then, the feature maps are sequentially passed through DSConv(16→32, k = 9) followed by MaxPool1d(5), and DSConv(32 → 48, k = 7) followed by MaxPool1d(5), reducing the temporal length to 250 and 50, respectively. Finally, DSConv(48 → 64, k = 5) is used to extract high-level features while keeping the temporal length at 50.

After the backbone network, global average pooling and global max pooling are applied in parallel, and their outputs are concatenated into a 128-dimensional global feature vector. During deployment and inference, this feature vector is passed only through a binary classification head, consisting of Linear(128 → 1) and sigmoid activation, to estimate the abnormality probability. The inference model contains approximately 6.4 k parameters, with exact statistics reported in Section 5. Therefore, from the perspective of parameter count and computational structure, it may be more suitable for potential microcontroller-level edge inference than standard convolutional models [47,48].

During training, an auxiliary multi-label classification head, Linear(128 → 5), is additionally introduced to fit the five-class diagnostic soft targets generated by the teacher model. This auxiliary head is used only in the distillation stage and is completely removed during inference, introducing no additional deployment cost. In this way, the student model benefits from richer multi-class diagnostic supervision during training while retaining a compact binary inference path for deployment.

### 3.4. Cross-Lead Knowledge Distillation Training Framework

**Figure 6:**
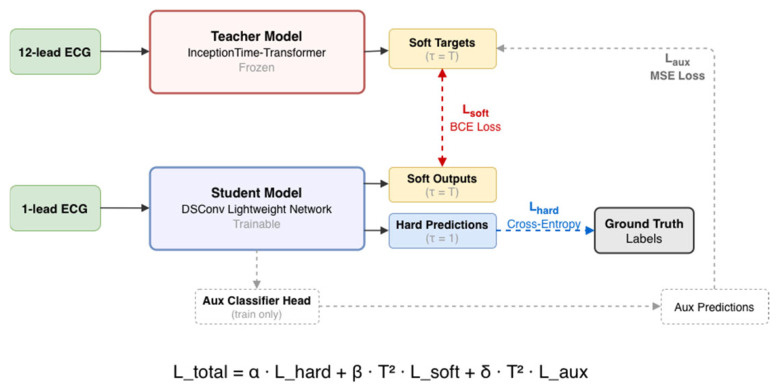
Overview of the hybrid knowledge distillation training pipeline. The frozen Teacher model generates temperature-scaled soft targets to supervise the Student model through three loss components: $L_{\mathrm{hard}}$, $L_{\mathrm{soft}}$, and $L_{\mathrm{aux}}$. The auxiliary multi-label head is discarded after training.

This section describes the complete distillation training process. As shown in Figure 6, the teacher model is frozen during the distillation stage, and only the student model is updated through backpropagation.

#### 3.4.1. Teacher Model Pre-Training

The teacher model is first trained independently on the 12-lead training set using five-class multi-label classification as the optimization objective. A weighted binary cross-entropy loss is used to address class imbalance. For the i-th diagnostic superclass, the positive class weight is defined as shown in Equation (3):

(3) $$w_{i}=\frac{N_{\mathrm{neg},i}}{N_{\mathrm{pos},i}}$$

where $N_{\mathrm{pos},i}$ and $N_{\mathrm{neg},i}$ denote the numbers of positive and negative samples for class i in the training set, respectively. These weights are automatically calculated from the class distribution and encourage the teacher model to pay more attention to minority classes. After convergence, all parameters of the teacher model are fixed, and the model is used as the knowledge provider in the subsequent distillation process.

#### 3.4.2. Loss Function Design

The total loss used in the distillation stage consists of three components: hard-label task loss, temperature-softened binary distillation loss, and auxiliary multi-label distillation loss.

1. Hard-Label Task Loss

The hard-label task loss is denoted as $L_{\mathrm{hard}}$. Let $z_{s}$ be the binary logit output by the student model. The binary ground-truth label is derived from the NORM label, as shown in Equation (4):

(4) $$y_{\mathrm{binary}}=1-y_{\mathrm{NORM}}$$

where $y_{\mathrm{NORM}}$ indicates whether the sample belongs to the normal class. The hard-label supervised loss is then calculated using weighted binary cross-entropy, as shown in Equation (5):

(5) $$L_{\mathrm{hard}}=\mathrm{BCE}!\left(\sigma \left(z_{s}\right),\;y_{\mathrm{binary}};\;w_{\mathrm{binary}}\right)$$

where σ(⋅) denotes the sigmoid function, and $w_{\mathrm{binary}}$ is the positive class weight for the binary abnormality classification task. This loss directly constrains the student model to learn a decision boundary consistent with the ground-truth binary labels.

2. Temperature-Softened Binary Distillation Loss

The temperature-softened binary distillation loss is denoted as $L_{\mathrm{soft}}$. Let $z_{t}^{\mathrm{NORM}}$ be the teacher logit corresponding to the NORM class. Since the student model performs binary normal–abnormal classification while the teacher model outputs five-class multi-label predictions, the teacher’s NORM output is converted into an abnormality soft label. As shown in Equation (6), the teacher-side temperature-scaled abnormality probability is defined as:

(6) $$p_{t}^{\mathrm{abn}}\left(T\right)=\sigma !\left(-\frac{z_{t}^{\mathrm{NORM}}}{T}\right)$$

where T is the temperature hyperparameter. The corresponding temperature-scaled output of the student model is defined in Equation (7):

(7) $$p_{s}\left(T\right)=\sigma !\left(\frac{z_{s}}{T}\right)$$

Accordingly, the temperature-softened binary distillation loss is formulated as shown in Equation (8):

(8) $$L_{\mathrm{soft}}=\mathrm{BCE}\left(\sigma \left(\frac{z_{s}}{T}\right),\;\sigma \left(-\frac{z_{t}^{\mathrm{NORM}}}{T}\right)\right)$$

When T > 1, the output distribution becomes smoother, which exposes finer-grained confidence information and allows the student model to learn the dark knowledge embedded in the teacher model [15,49].

3. Auxiliary Multi-Label Distillation Loss

To further transfer the multi-label diagnostic knowledge learned by the teacher model from 12-lead ECG inputs, an auxiliary multi-label output head is introduced into the student network. Let the five-dimensional auxiliary logit vector of the student model be defined as shown in Equation (9):

(9) $$z_{s}^{aux}\in R^{5}$$

and let the teacher output logit vector be defined as shown in Equation (10):

(10) $$z_{t}\in R^{5}$$

After temperature scaling and sigmoid mapping, mean squared error is used to measure the difference between the teacher and student multi-label soft probabilities. The auxiliary multi-label distillation loss is defined in Equation (11):

(11) $$L_{\mathrm{aux}}=\mathrm{MSE}\left(\sigma \left(\frac{z_{s}^{aux}}{T}\right),\;\sigma \left(\frac{z_{t}}{T}\right)\right)$$

This loss encourages the student backbone to learn fine-grained diagnostic representations formed by the teacher model in the multi-lead input space, thereby enhancing the representational capacity of the single-lead student model.

4. Total Loss Function

The total distillation loss is computed as a weighted sum of the three components, as shown in Equation (12):

(12) $$L_{\mathrm{total}}=\alpha L_{\mathrm{hard}}+\beta T^{2}L_{\mathrm{soft}}+\delta T^{2}L_{\mathrm{aux}}$$

where α, β, and δ denote the weighting coefficients of the three loss terms. The factor $T^{2}$ is used to compensate for the gradient magnitude reduction caused by temperature scaling [15]. In this study, the hyperparameters are set as shown in Equation (13):

(13) α=0.3          β=0.5          δ=0.3          T=4

Note that the three coefficients are not required to sum to unity, as the T^2^ scaling factors on $L_{\mathrm{soft}}$ and $L_{\mathrm{aux}}$ already modulate the effective gradient magnitudes. The relatively large value of β reflects the emphasis of the proposed framework on teacher soft-label supervision. In the cross-lead extreme compression scenario, confidence distribution information conveyed by soft labels is particularly important for compensating for information loss caused by single-lead input.

### 3.5. Training Strategy

Both the teacher and student models are optimized using AdamW, with the weight decay coefficient set to $1\times 10^{-4}$ [50]. The learning rate is scheduled using cosine annealing [51] with a 10-epoch linear warm-up. The initial learning rate of the teacher model is set to $1\times 10^{-3}$, and the maximum number of training epochs is 150. The initial learning rate of the student model is set to $2\times 10^{-3}$, and the maximum number of training epochs is 120.

Early stopping is applied based on the Macro-F1 score on the validation set, with the patience value set to 25 epochs for both models. During training, automatic mixed precision is enabled to improve computational efficiency [52]. Gradient clipping is also used, with the maximum gradient norm set to 1.0 [53].

During teacher model training, lightweight online data augmentation is applied to improve generalization and the quality of teacher-generated soft labels. Specifically, Gaussian noise with a standard deviation of 0.015 is added to the ECG signals, and random amplitude scaling within ±8% is applied for each training batch. These operations follow common ECG data augmentation practices [54], and the specific parameters are determined through preliminary experiments.

During inference, the default classification threshold of 0.5 is not directly used. Instead, the decision threshold is searched on the validation set from 0.20 to 0.80 with a step size of 0.01, and the threshold maximizing Macro-F1 is used for test-set evaluation.

To ensure statistical reliability, all training and evaluation procedures are repeated using five random seeds. The final results are reported as mean ± standard deviation. Detailed experimental configurations and results are presented in Section 5.

## 4. System Design and Implementation

### 4.1. Problem Analysis and Design Approach

To address the system-level limitations identified in Section 2.5, namely the lack of a structured mechanism for integrating cloud-based large language models with on-device preliminary screening results and real-time physiological indicators, and the absence of a closed-loop collaboration framework between lightweight edge models and cloud-based large models, this section presents the design and implementation of the proposed edge–cloud collaborative ECG analysis system. Specifically, this section introduces the overall system architecture, the cloud-based large language model service, and the WeChat Mini Program implementation.

### 4.2. Overall System Architecture

**Figure 7:**
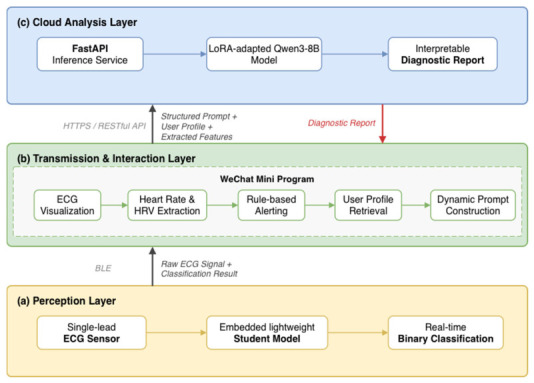
Overall architecture of the three-layer edge–cloud collaborative ECG system: perception layer (ECG sensor + embedded student model), transmission and interaction layer (WeChat Mini Program), and cloud analysis layer (LoRA-fine-tuned Qwen3-8B).

As shown in Figure 7, the proposed system adopts a three-layer edge–cloud collaborative architecture [55], consisting of the perception layer, the transmission and interaction layer, and the cloud-based analysis layer.

The perception layer is responsible for ECG signal acquisition and real-time on-device screening. A portable single-lead ECG sensor transmits the acquired ECG signals to the mobile terminal via Bluetooth Low Energy (BLE) [56], and an embedded microcontroller is expected to run the lightweight student model described in Section 3 for binary normal–abnormal screening. In the current prototype, however, the on-device model output is simulated using internal variables within the Mini Program, and end-to-end embedded deployment, including BLE latency measurement and physical-device integration, will be completed in future work.

The transmission and interaction layer is implemented based on the WeChat Mini Program platform [57]. This layer serves as the intermediate component for data relay, user interaction, and lightweight front-end computation. It receives raw ECG signals and preliminary classification results from the perception layer, visualizes the ECG waveform, calculates key physiological indicators, and generates rule-based local alerts. In addition, it organizes the on-device screening result, real-time physiological features, and user history into a structured prompt, which is then submitted to the cloud inference service via HTTPS.

The cloud analysis layer deploys a Qwen3-8B large language model [18] fine-tuned using LoRA [19], implemented with the PEFT library (v0.18.0). After receiving the structured prompt, the cloud service integrates the preliminary screening result from the edge device with the user’s background information and real-time physiological indicators. Following a structured reasoning paradigm inspired by Chain-of-Thought prompting [58], the model generates an interpretable health analysis report and returns it to the Mini Program for display.

### 4.3. Cloud-Based Large Language Model Service

#### 4.3.1. Selection of the Foundation Model and LoRA Fine-Tuning

To enable the system to organize and present ECG-related indicators, user background information, and preliminary screening results in a structured report format, this study introduces a large language model as the cloud-side report generation engine. Considering Chinese medical text comprehension, reasoning ability, and deployment cost, Qwen3-8B [18] was selected as the foundation model.

Given that the experimental deployment environment is equipped with an NVIDIA GeForce RTX 5070 Ti GPU with 16 GB of VRAM (Nvidia Corporation, Santa Clara, CA, USA), a 4-bit quantization strategy [59] was adopted during model loading to reduce memory consumption and inference cost. This configuration enables the 8B-scale model to be loaded and executed on a single consumer-grade GPU.

To adapt the general-purpose large language model to cardiovascular-oriented analysis tasks, this study employs Low-Rank Adaptation (LoRA) [19] for parameter-efficient fine-tuning. LoRA freezes the original pre-trained weight matrix and introduces a low-rank trainable update. As shown in Equation (14), the updated weight matrix can be expressed as:

(14) $$W=W_{0}+\Delta W=W_{0}+BA$$

where $W_{0}$ denotes the frozen pre-trained weight matrix, $B\in R^{d\times r}$ and $A\in R^{r\times k}$ are trainable low-rank matrices, and r≪min(d,k). In this implementation, LoRA adapters are injected into the query, key, value, and output projection layers of the Transformer architecture, namely q_proj, k_proj, v_proj, and o_proj, as well as the feed-forward network modules gate_proj, up_proj, and down_proj.

The key fine-tuning hyperparameters are summarized in Table 1. Under this configuration, the number of trainable parameters is approximately $1.746\times 10^{8}$, accounting for about 2.09% of the total model parameters, demonstrating the parameter efficiency of the fine-tuning strategy.

To ensure that the model possesses both general cardiovascular reasoning ability and adaptability to the system deployment format, this study adopts a two-stage data construction strategy and mixes the two types of data for supervised fine-tuning.

The first stage constructs domain knowledge expansion data. Based on the publicly available medical reasoning dataset medical-o1-reasoning-SFT [60], cardiovascular-related core question–answer samples were selected. Subsequently, DeepSeek-V3 was used to expand these samples by generating outputs containing structured intermediate reasoning and complete medical responses. This process was intended to improve the reasoning depth and response completeness of the training samples.

The second stage constructs deployment-oriented synthetic data. To reduce the distribution gap between general medical question answering and actual system inputs, this study generated synthetic samples aligned with the structured prompts used in the WeChat Mini Program. Synthetic samples were constructed by combining user profiles, ECG-related physiological indicators, edge-side screening results, and chief complaint templates, with medically unreasonable combinations filtered by predefined rules. DeepSeek-V3 was then used to generate corresponding analysis reports. Since these reports were generated by an LLM rather than written by clinicians, the resulting synthetic data may contain generator-dependent biases or distributional artifacts. In this study, the synthetic samples were used primarily to align the fine-tuned model with the structured input–output format of the prototype system, rather than as a substitute for real clinical report supervision.

Finally, the two types of data were merged and randomly shuffled to form a hybrid fine-tuning dataset containing approximately 6355 training samples. Multi-level quality control—including format, length, and consistency checks—was applied, and approximately 10% of samples were additionally reviewed manually. Nevertheless, fine-grained biases may still remain in the synthetic data.

Each training sample consists of three fields: Clinical Question, Intermediate Analysis Process, and Final Answer, allowing the model to learn a structured mapping from deployment-style ECG inputs to interpretable health reports.

**Figure 8:**
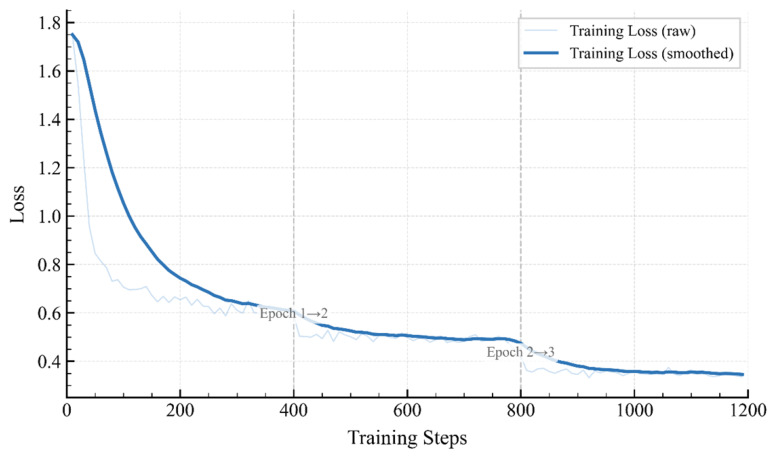
Training loss curve during large language model fine-tuning. The shaded curve represents the original mini-batch loss, while the solid line denotes the smoothed loss obtained by moving average. The overall downward trend indicates stable convergence during training.

The training process was implemented using the Unsloth acceleration framework (v2025.11.4) [61] and the SFTTrainer module in TRL (v0.24.0) [62]. Training was conducted on a single RTX 5070 Ti GPU, with gradient checkpointing and VRAM offloading enabled to control peak memory usage. The model was trained for 1194 steps, corresponding to approximately three epochs. As shown in Figure 8, the training loss decreased from approximately 1.75 to 0.34, showing a generally smooth downward trend without significant oscillation or divergence.

#### 4.3.2. Inference Service Construction and Deployment

The cloud-based inference service is implemented using the FastAPI framework (v0.122.0) [63], which provides a /chat endpoint to receive POST requests from the Mini Program. When the service starts, it loads the fine-tuned Qwen3-8B model and the corresponding tokenizer. After receiving a JSON request containing the query field, the service inserts the user input into the predefined inference template and calls the autoregressive generation interface of the model to generate the response.

During inference, the generation temperature is set to 0.7 to balance response stability and linguistic naturalness, while max_new_tokens is set to 1024 to control the maximum output length. After generation, the server extracts the content following the </think> tag as the final response and returns it to the Mini Program in JSON format.

### 4.4. Design and Implementation of the WeChat Mini Program

#### 4.4.1. ECG Signal Visualization and Physiological Parameter Extraction

The WeChat Mini Program is responsible for front-end display and lightweight signal processing. After receiving ECG data, the Mini Program uses the Canvas 2D API to draw the ECG waveform and overlays a grid background to approximate the visual style of a clinical ECG chart. The interface also displays the time axis, amplitude axis, and key physiological indicators, allowing users to quickly understand the current monitoring results.

For feature extraction, the system implements an R-wave detection method based on thresholding and local maximum criteria. R-wave peaks are detected using a threshold-based local maximum method, which is lightweight enough for real-time execution in the Mini Program’s JavaScript environment.

Based on the detected R-wave peak sequence, the system further calculates the average heart rate and the heart rate variability metric SDNN. Specifically, the differences between adjacent R-peak indices are converted into RR intervals according to the sampling rate. The average heart rate (unit: beats per minute) is calculated as Equation (15):

(15) $${HR}_{avg}=\frac{60}{\bar{RR}}$$

Heart rate variability is quantified by the standard deviation of normal-to-normal intervals (SDNN), defined in Equation (16):

(16) $$SDNN=\sqrt{\frac{1}{N-1}\sum_{i=1}^{N}{\left(NN_{i}-\bar{NN}\right)}^{2}}$$

respectively. These two indicators are then used for real-time physiological status assessment and subsequent prompt construction.

Based on these indicators, a rule-driven alert mechanism classifies the status into normal, warning, and critical levels using clinically referenced thresholds.

#### 4.4.2. Dynamic Prompt Construction and Edge–Cloud Interaction

After the user activates the “AI Deep Analysis” function, the Mini Program first retrieves the user’s basic information, including height, weight, age, gender, and medical history, from the cloud database. It then integrates this static background information with the current ECG-related physiological indicators and the preliminary screening result from the edge model into a structured natural language input [64], which is submitted to the cloud inference service.

The core idea of this strategy is to embed the on-device classification result into the context of the large language model as prior information, thereby guiding the cloud-side model to adopt differentiated analysis strategies under different risk conditions. When the on-device result is “abnormal”, an emphasized prefix is added to the prompt to guide the model to focus on potential pathological risks, such as myocardial ischemia, myocardial infarction, or conduction abnormalities. When the on-device result is “normal”, a standard descriptive template is used. Through this design, the system converts rapid edge-side screening results into contextual constraints for cloud-side inference, improving the consistency between the generated report and the on-device judgment. As a result, patients with the same binary screening label may still receive different LLM-generated interpretations and recommendations because the structured prompt also contains patient-specific context, including demographic information, medical history, body mass index (BMI), heart rate, and SDNN.

**Figure 9:**
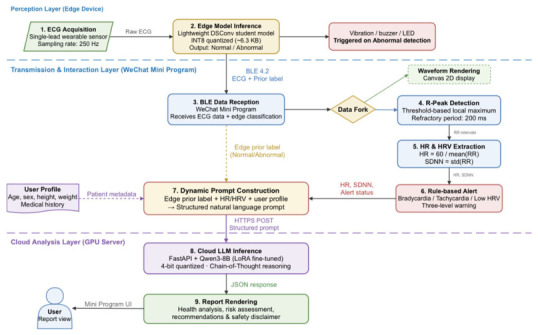
End-to-end workflow of the proposed edge–cloud collaborative ECG-assisted analysis system. The pipeline spans three layers: the perception layer performs ECG acquisition and lightweight on-device inference; the transmission and interaction layer handles BLE data reception, physiological feature extraction, rule-based alerting, and dynamic prompt construction through the WeChat Mini Program; and the cloud analysis layer generates personalized health reports using the LoRA-fine-tuned Qwen3-8B large language model.

The constructed prompt is submitted to the cloud interface via HTTPS, with a request timeout of 120 s to accommodate the latency of large model inference. After the server returns the generated analysis text, the Mini Program renders the result on the analysis page. This completes the prototype-level closed-loop process from signal acquisition, edge screening output, feature extraction, prompt construction, cloud-side generation, and result feedback. As shown in Figure 9, the proposed workflow illustrates a potential implementation path for combining on-device perception with cloud-based semantic analysis, the practical viability of which requires further validation on physical hardware.

#### 4.4.3. Example of Cloud-Side Generation Results

To illustrate the end-to-end output format of the proposed system, a representative structured input case is summarized here. The input describes a 45-year-old male user with a history of hypertension, an average heart rate of 95 bpm, an SDNN of 45.2 ms, and an edge-side screening result of “abnormal”.

Table 2 presents a condensed English translation of the Chinese report generated by the fine-tuned Qwen3-8B model. The excerpt illustrates the main output sections, including risk assessment, ECG-related indicator interpretation, possible risk directions, and follow-up recommendations. The report correctly computes the BMI as 22.9 kg/m^2^, incorporates the hypertension history, references the heart rate and SDNN values, and maintains a cautious auxiliary-reference tone rather than presenting a diagnostic conclusion. Quantitative evaluation of report quality is provided in Section 5.8.

## 5. Experiments and Results Analysis

### 5.1. Experimental Setup

All experiments were conducted on a workstation equipped with an NVIDIA GeForce RTX 5070 Ti GPU with 16 GB of VRAM under the Windows operating system. The software environment included Python 3.10.20, PyTorch 2.10.0, CUDA 12.8, and scikit-learn 1.7.2. To improve reproducibility, all stochastic operations were controlled by fixed random seeds, including torch.manual_seed, np.random.seed, and torch.cuda.manual_seed_all. In addition, cuDNN benchmark mode was disabled, and deterministic mode was enabled.

#### 5.1.1. Dataset Splitting and Statistics

The experiments were conducted on the PTB-XL dataset [39]. The original recordings consist of 12-lead ECG signals sampled at 500 Hz. After preprocessing, all signals were downsampled to 250 Hz. The dataset split and class distribution are summarized in Table 3.

Following the official patient-level stratified split of PTB-XL, folds 1–8 were used for training, fold 9 for validation, and fold 10 for testing [40]. This fold assignment ensures that all recordings from the same patient are allocated exclusively to a single fold; consequently, the training, validation, and test sets comprise entirely non-overlapping patient groups, preventing potential data leakage across subsets.

#### 5.1.2. Evaluation Metrics

Accuracy, Macro-F1, and AUC-ROC were adopted as the main evaluation metrics. Macro-F1 was used as the primary metric because it gives equal importance to normal and abnormal classes and is therefore more suitable for evaluating performance under class imbalance. AUC-ROC was used to assess the threshold-independent discriminative ability of the model [39].

The binary classification threshold was optimized on the validation set, as described in Section 3. The main experiments were repeated using five random seeds, namely 42, 123, 456, 789, and 1024, and the results are reported as mean ± standard deviation. Ablation studies and comparisons with lightweight baselines were conducted using three random seeds, namely 42, 123, and 456, to balance computational cost and statistical reliability [65].

### 5.2. Five-Class Diagnostic Performance of the Teacher Model

Before performing cross-lead knowledge distillation, the diagnostic capability of the teacher model was first evaluated on the five-class diagnostic superclass task. This evaluation was intended to verify whether the teacher model could provide sufficiently informative soft-label supervision for subsequent student training. Table 4 reports the per-class performance of the teacher model on the test set.

The teacher model achieved AUC values above 0.89 for all five diagnostic superclasses, indicating a strong overall discriminative capability in the multi-label setting.

**Figure 10:**
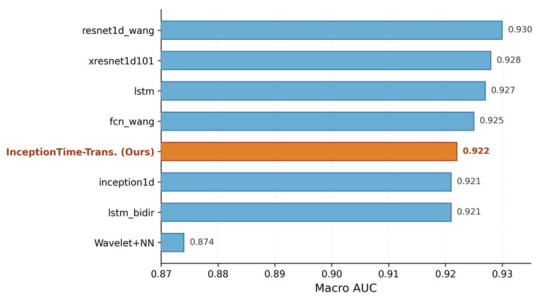
Macro AUC comparison between the proposed teacher model and representative baselines on the PTB-XL diagnostic superclass benchmark. Baseline results are taken from the PTB-XL public benchmark [40]. The proposed InceptionTime-Transformer teacher model achieves a Macro AUC of 0.922, supporting its use as the knowledge source for downstream cross-lead distillation.

To further contextualize the teacher model’s performance, Figure 10 compares its Macro AUC with representative models reported in the public PTB-XL diagnostic superclass benchmark [40]. As differences in preprocessing and training protocols may exist across studies, this comparison serves as a reference for performance positioning rather than a strictly controlled evaluation. The proposed InceptionTime–Transformer teacher achieved a Macro AUC of 0.922, outperforming the representative CNN-based and LSTM-based baselines included in the PTB-XL benchmark comparison. Although this benchmark comparison does not constitute a full distillation-teacher ablation, it indicates that the hybrid teacher provides a strong 12-lead diagnostic model and supports its selection as the source of soft-label supervision under the extreme compression and lead-reduction constraints of this study.

### 5.3. Main Results of Cross-Lead Knowledge Distillation

Table 5 summarizes the binary classification performance of the teacher model and the distilled student model on the test set. The teacher model uses 12-lead ECG signals as input, whereas the student model only uses single-lead Lead II signals. All results are reported as mean ± standard deviation over five random seeds.

**Figure 11:**
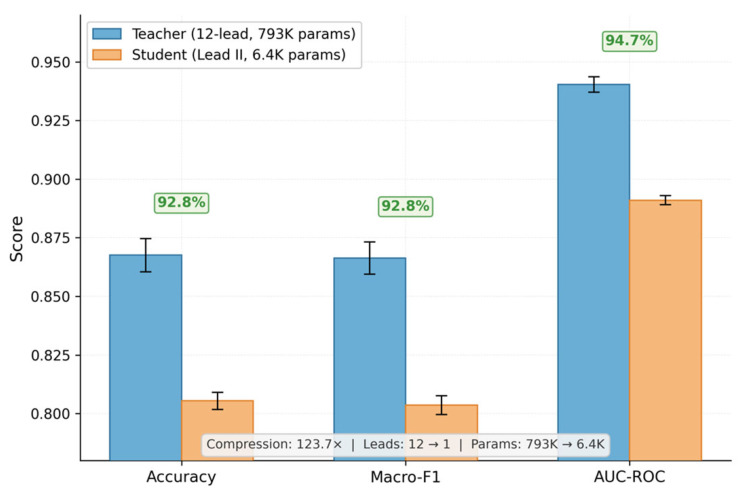
Binary classification performance comparison between the teacher model and the student model on the PTB-XL test set. The teacher model uses 12-lead ECG signals and contains 793,733 inference parameters, whereas the student model uses single-lead Lead II signals and contains 6417 inference parameters. Results are reported as mean ± standard deviation over five random seeds. The student model retains 92.8% of the teacher model’s Macro-F1 and 94.7% of its AUC-ROC under 123.7× parameter compression and 12-to-1 lead reduction.

As shown in Table 5 and visualized in Figure 11, the student model retained 92.8% of the teacher’s Macro-F1 and 94.7% of its AUC-ROC despite simultaneous 12-to-1 lead reduction and 123.7× parameter compression, suggesting that the proposed distillation strategy partially mitigates degradation under extreme constraints [29,49]. The low cross-seed AUC-ROC variance (std = 0.0019) further indicates stable ranking performance desirable for edge-oriented screening.

### 5.4. Comparison with Lightweight Models of Similar Parameter Scale

To evaluate the competitiveness of the proposed student model under a comparable resource budget, two lightweight baselines with similar parameter counts were introduced: TinyCNN, consisting of three standard 1D convolutional layers, and TinyResNet, consisting of two residual blocks. Both baselines use single-lead Lead II ECG signals as input and are trained with the same optimization settings as the student model, but only with hard-label supervision. In addition, a non-distilled DSConv model, denoted as DSConv(Direct), was included to isolate the contribution of the distillation strategy.

As shown in Table 6, Accuracy and Macro-F1 differences among all four models are small and largely within one standard deviation, suggesting that hard-label performance is near the practical upper bound of this resource setting. Nevertheless, DSConv(KD) achieves the highest AUC-ROC (0.8906), indicating that teacher soft-label supervision improves probability ranking quality—a property useful for edge screening where the operating threshold may need adjustment. Compared with TinyResNet, which has 18.4% more parameters, DSConv(KD) offers a more favorable trade-off between discriminative performance and parameter efficiency [66].

### 5.5. Ablation Studies

To further examine the contribution of each key design choice in the proposed distillation framework, we conducted ablation studies from three perspectives: loss component design, distillation temperature, and input lead selection. All ablation experiments were repeated using three random seeds, and the results are reported as mean ± standard deviation.

#### 5.5.1. Loss Component Ablation: Experiment A

Table 7 reports the test-set performance of the student model under different loss configurations.

Under single-lead ultra-lightweight constraints, absolute differences among configurations are small, indicating that performance is largely bounded by available signal information. Teacher supervision generally maintains or improves AUC-ROC, while the Full configuration achieves the lowest AUC-ROC standard deviation (0.0003), indicating the most reproducible training behavior across seeds; it is therefore adopted as the default. A supplementary sweep over distillation temperatures (T ∈ {1, 2, 4, 8, 16}) yielded only marginal performance differences; T = 4 was selected because it showed the lowest cross-seed AUC-ROC variance.

#### 5.5.2. Lead Selection Ablation: Experiment B

Table 8 reports the results obtained when three representative limb leads are used as the input of the student model.

Lead II achieves the best performance and lowest variance across all metrics, consistent with its closer alignment to the main cardiac electrical axis [46]. Lead III performs substantially worse, confirming that lead selection directly affects the amount of useful information available under single-lead constraints.

### 5.6. Model Efficiency Analysis

To evaluate the edge-deployment potential of the proposed student model, we further compare the teacher model and the student inference model in terms of parameter count, model size, simulated inference latency, and memory footprint.

As shown in Table 9, the student model contains only 6417 inference parameters. Even under FP32 precision, its weight size is approximately 25.1 KB; after INT8 quantization, the pure parameter size is further reduced to approximately 6.3 KB. On the GPU platform, the student model achieves an inference latency of 0.38 ms, which is approximately 6.4 times faster than the teacher model.

To further assess whether the model size and runtime are compatible with microcontroller-class resource constraints, the INT8-quantized student model was exported to TensorFlow Lite Micro (TFLM) format and evaluated on the ARM Cortex-M4 architecture using the QEMU (v10.2.2) [67] simulator with the MPS2-AN385 simulation platform [68]. The resulting quantized TFLite model file is 20.8 KB, including the computational graph structure and quantization parameters. The Tensor Arena memory usage reported by the TFLM runtime is 63.0 KB, which falls within the 192 KB on-chip SRAM capacity of the STM32F407 in simulation.

The total estimated inference time is 11.6 ms at 168 MHz. This latency is far below the 10 s ECG acquisition window, accounting for only approximately 0.12% of the window duration. These simulation-based results suggest that the proposed student model satisfies the storage and computational constraints of a typical Cortex-M4 microcontroller; however, actual deployment feasibility requires further validation on physical hardware, including assessment of power consumption, peripheral integration, and real-time scheduling overhead [69].

It should be noted that QEMU provides instruction-level simulation results, which may differ from actual hardware performance due to factors such as Flash wait states, memory access behavior, and cache effects. Therefore, real-hardware validation will be conducted in future work.

### 5.7. Cloud-Side Large Language Model Evaluation Setup

To evaluate the analysis and report-generation capability of the cloud-side large language model in structured ECG scenarios, experiments were conducted using the domain-adapted Qwen3-8B model described in Section 4.3.

The evaluation adopts the same structured prompt template used in the prototype system, integrating edge-side screening results, user demographics, heart rate, and HRV.

To balance generation stability and linguistic naturalness, the default decoding parameters are set as follows: temperature = 0.7, top_p = 0.9, and repetition_penalty = 1.1. In online service scenarios, max_new_tokens is set to 1024 to control response length and inference latency. For offline case analysis and comparative evaluation, a larger output limit is used to preserve the completeness of the generated report structure.

The large-language-model experiments were conducted using Unsloth 2025.11.4, Transformers 4.57.1, TRL 0.24.0, PEFT 0.18.0, and CUDA 12.8. The evaluation does not aim to measure general language generation ability. Instead, it focuses on four system-oriented aspects: whether the model can adapt to the structured ECG input format, whether it can correctly reference key physiological indicators and user background information, whether its conclusion remains consistent with the edge-side screening result, and whether the generated content is safe, appropriately cautious, and formatted in a manner suitable for auxiliary health reference rather than independent clinical decision-making [31].

### 5.8. Comparative Evaluation of the Fine-Tuned and Base Models

To examine whether domain-specific fine-tuning improves the model’s adaptability to the proposed ECG-assisted analysis task, we compared the fine-tuned Qwen3-8B model, denoted as ***Ours-FT***, with the original unfine-tuned Qwen3-8B base model, denoted as ***Base***. Both models shared the same backbone architecture, 4-bit quantization scheme, input template, and inference settings. Therefore, the observed differences can be mainly attributed to the domain-specific data construction strategy and LoRA-based fine-tuning.

The evaluation combined blind human assessment with automated auxiliary metrics. For the manual evaluation, three medical evaluators with clinical experience in cardiology independently scored the generated reports under blinded conditions. The manual test set contained six representative cases spanning normal and abnormal screening results, varied age groups, risk profiles, and ECG indicator combinations. Given this limited scale, the results serve as preliminary evidence of task adaptability rather than large-scale clinical validation. For each case, both models generated reports from the same structured input under blinded conditions (identifiers removed, outputs randomly numbered). Five dimensions—medical accuracy, structural completeness, end-to-end consistency, personalization, and safety compliance—were each rated on a 1–5 scale [64]. Results are summarized in Table 10.

As shown in Table 10, Ours-FT outperformed the Base model across all five evaluation dimensions. The improvement was particularly evident in structural completeness, end-to-end consistency, and personalization, indicating that domain-specific fine-tuning helped the model better follow the expected report format, align its analysis with edge-side screening information, and incorporate patient-specific background factors. The reduced standard deviation of the overall score also suggests that fine-tuning improved the stability of the generated reports across different cases. Nevertheless, because the manual evaluation was conducted on a small set of representative cases, these results should be interpreted as preliminary evidence of task-oriented report generation rather than definitive clinical validation. The evaluation focused on report structure, consistency with the structured input, personalization, and safety-oriented expression, but did not assess clinical diagnostic accuracy against real clinician-written reports.

In addition to manual scoring, automated auxiliary metrics were introduced to provide a broader-scale supplementary evaluation. An additional 94 cases were constructed from PTB-XL test-fold records, resulting in a 100-case automated evaluation set covering all five diagnostic superclasses. This design aimed to evaluate whether the model could respond appropriately to variations in both diagnostic context and physiological indicators.

To further distinguish the effect of domain-specific fine-tuning from that of edge-side prior embedding, three experimental conditions were evaluated on the same automated test set: (1) Base model with edge-side prior, (2) Ours-FT model with edge-side prior, and (3) Ours-FT model without edge-side prior. Conditions (1) and (2) were used to evaluate the contribution of LoRA fine-tuning, whereas conditions (2) and (3) formed an ablation comparison for the edge-side prior. In the latter comparison, the same fine-tuned model was used, and the only difference was whether the input prompt contained the edge-side preliminary screening result. The results are reported in Table 11. Here, section coverage refers to the presence of six predefined task-relevant elements in the generated report: heart rate, SDNN, edge-side screening result, health advice, risk explanation, and medical consultation suggestion.

As shown in Table 11, domain-specific fine-tuning reduced the average report length from 5081 to 2329 characters while preserving section coverage (93.0% → 92.5%), indicating more concise yet structurally complete outputs. BMI MAE decreased from 1.10 to 0.38, suggesting improved numerical consistency. When the edge-side prior was removed, section coverage decreased from 92.5% to 86.2%, suggesting that the prior helps guide the model to include task-relevant report elements. These automated results are consistent with the manual assessment in Table 10 and provide reproducible supplementary evidence for evaluating structural standardization, numerical consistency, and task alignment.

### 5.9. Discussion on Experimental Validity

Although the edge-side evaluation was conducted in QEMU [67] rather than on physical hardware, QEMU is a mature open-source emulator widely used for early-stage embedded and TinyML validation [70], and the present simulation examined model storage, SRAM occupancy, and inference latency under Cortex-M4-class constraints. Therefore, while real-device testing remains necessary, the simulation results still provide meaningful preliminary evidence and practical guidance for subsequent hardware deployment. For the cloud-side LLM, the fine-tuning data were used for format adaptation rather than clinical supervision; nevertheless, the first-stage data were grounded in a public medical reasoning dataset (medical-o1-reasoning-SFT [60]), the second-stage samples were constrained by prototype-specific structured inputs and plausibility filtering, and the final evaluation was conducted on 100 cases constructed from real PTB-XL test-fold recordings. These results suggest that the proposed framework has credible preliminary validity, although further validation with physical devices and clinician-written ECG reports remains future work.

## 6. Conclusions and Future Directions

This paper proposed an edge–cloud collaborative ECG analysis method that integrates cross-lead knowledge distillation with large language model-assisted cloud analysis, and examined its technical viability through a proof-of-concept prototype system under simulated conditions. At the algorithmic level, a hybrid distillation objective was designed by combining hard-label supervision, temperature-smoothed soft-label distillation, and auxiliary multi-label distillation. This enabled diagnostic knowledge learned from a 12-lead teacher model to be transferred to an ultra-lightweight single-lead student model. Under a 123.7× parameter compression ratio and a 12-to-1 lead reduction, the student model retained 92.8% of the teacher model’s Macro-F1 performance, suggesting that discriminative ECG knowledge can be substantially preserved under highly constrained edge-device conditions in simulation. At the system level, a three-tier collaborative framework was developed to connect on-device preliminary screening with cloud-based deep analysis. A LoRA-fine-tuned Qwen3-8B model was further adapted to structured ECG-related inputs, enabling the system to generate structured auxiliary health reports that reference edge-side screening results, physiological indicators, and user background information.

Despite these preliminary results, several limitations remain. First, the current edge-deployment evaluation is based on QEMU instruction-level simulation rather than end-to-end testing on physical microcontroller hardware. Consequently, the model’s actual inference latency, energy consumption, BLE communication latency, motion-artifact robustness, and operational stability when integrated with peripheral modules on physical hardware remain to be verified. Second, the cloud-side inference service is currently implemented as a prototype deployment. Its long-term operational stability, network latency, and concurrent service capacity remain insufficient for large-scale real-world use. Third, the experiments were mainly conducted on the public PTB-XL dataset, which was collected using clinical-grade devices under relatively controlled conditions. In contrast, wearable single-lead ECG signals collected during daily activities may exhibit different noise patterns, signal quality, and spectral characteristics. Consequently, the cross-domain generalization ability of the proposed method requires further validation. Moreover, the student model is currently validated only on standard Lead II; its generalization to other standard leads or non-standard leads from consumer wearable devices remains untested and may require lead-specific calibration or retraining, which constitutes an important direction for future work. In addition, part of the cloud-side training data was generated synthetically based on predefined parameter spaces. Although such data help align the model output format with the target deployment workflow, they do not substitute for real clinical data. Because no real clinical report corpus was used for supervised fine-tuning, the current results should be interpreted as evidence of structured report generation within the prototype system rather than clinically validated diagnostic capability. Future work should incorporate clinician-written ECG reports or expert-curated clinical datasets collected under appropriate ethical approval, informed consent, and data de-identification procedures to improve the representativeness and credibility of the training distribution. Finally, the reports generated by the cloud-side large language model are intended only for auxiliary health analysis and early warning, rather than as independent clinical diagnostic evidence. Issues related to medical reliability, safety, liability, and regulatory compliance must be examined under stricter clinical validation and ethical review frameworks before practical deployment.

Future work will proceed in four directions. First, the INT8-quantized student model will be deployed on physical MCU platforms (e.g., STM32F407) to validate simulation results and evaluate power consumption, quantify BLE transmission latency, and evaluate robustness under motion artifacts and ambulatory signal noise. Second, the cloud inference service will be migrated to a production-grade GPU environment for improved stability and scalability. Third, real single-lead ECG data from wearable devices will be incorporated, together with domain adaptation strategies, to improve cross-domain robustness. Finally, a retrieval-augmented generation mechanism backed by a structured cardiovascular knowledge base will be introduced to enhance the factual consistency and clinical reference value of the generated reports.

## Figures and Tables

**Table 1:** Fine-tuning hyperparameter settings for the large language model.

Hyperparameter	Value
LoRA rank r	64
LoRA scaling factor α	128
LoRA dropout	0.05
Attention target modules	q_proj, k_proj, v_proj, o_proj
MLP target modules	gate_proj, up_proj, down_proj
Trainable parameters	174,587,904, 2.09%
Effective batch size	16, 1 × 16
Training epochs/steps	3 epochs, 1194 steps
Warm-up ratio	0.1
Learning rate	$2\times 10^{-4}$
Learning rate schedule	Cosine annealing
Optimizer	AdamW-8bit
Weight decay	0.01
Maximum sequence length	4096 tokens
Precision	BFloat16

**Table 2:** Condensed excerpt from the cloud-side generated report for a representative input case, translated from Chinese.

Report Section	Representative Content (Translated Excerpt)
BMI Assessment	BMI is calculated as 22.9 kg/m^2^, within the normal range; obesity is not identified as an additional risk factor.
Risk Factors	The user is male, 45 years old, and has a 5-year history of hypertension, which is identified as the primary cardiovascular risk factor.
Heart Rate	The average heart rate is 95 bpm, near the upper limit of the normal range, possibly reflecting increased cardiac workload or sympathetic activation.
HRV Analysis	SDNN is 45.2 ms, below the 50 ms short-term reference threshold, suggesting reduced autonomic regulation consistent with chronic hypertension.
Risk Directions	Based on the edge-side abnormal screening result, hypertension history, high-normal heart rate, and reduced HRV, myocardial ischemia, hypertensive cardiac remodeling, and non-specific ST-T changes are discussed as possible explanations, but not as confirmed diagnoses.
Recommendations	Standard 12-lead ECG, echocardiography, and metabolic blood testing are recommended. The report states that the AI screening result and generated analysis are for auxiliary reference only and cannot replace clinical diagnosis.

Note: The original report was generated in Chinese. This table presents condensed English translations of representative statements from the generated report.

**Table 3:** PTB-XL dataset split and positive sample statistics for each diagnostic superclass.

Data Subset	Total Samples	NORM	MI	STTC	HYP	CD
Training set	17,100	7274	4389	4094	2121	3912
Validation set	2155	920	544	521	271	497
Test set	2162	917	553	508	263	498

**Table 4:** Five-class diagnostic superclass classification performance of the teacher model on the test set.

Category	AUC	AP	F1	Acc	Precision	Recall	Positive Samples
NORM	0.9398	0.9072	0.8476	0.8649	0.8128	0.8855	917
MI	0.9244	0.8356	0.7537	0.8760	0.7664	0.7414	553
STTC	0.9314	0.8186	0.7711	0.8858	0.7285	0.8189	508
HYP	0.8989	0.6634	0.6170	0.9001	0.5781	0.6616	263
CD	0.9177	0.8419	0.7613	0.8950	0.7991	0.7269	498
Macro avg.	0.9224	—	0.7501	—	—	—	—

**Table 5:** Binary classification performance comparison between the teacher and student models on the test set.

Model	Input	Inference Parameters	Accuracy	Macro-F1	AUC-ROC
Teacher	12 leads	793,733	0.8676 ± 0.0071	0.8663 ± 0.0069	0.9404 ± 0.0033
Student	Lead II	6417	0.8054 ± 0.0037	0.8036 ± 0.0040	0.8910 ± 0.0019
Retention	—	—	92.8%	92.8%	94.7%

Note: Teacher refers to the InceptionTime-Transformer model, and Student refers to the DSConv model. The student model achieves a 123.7× reduction in inference parameters compared with the teacher model.

**Table 6:** Comparison with lightweight models of similar parameter scale on the test set.

Model	Parameter Count	Accuracy	Macro-F1	AUC-ROC
TinyCNN	6437	0.8016 ± 0.0041	0.7999 ± 0.0032	0.8850 ± 0.0025
TinyResNet	7601	0.8039 ± 0.0033	0.8021 ± 0.0038	0.8871 ± 0.0012
DSConv(Direct)	6417	0.8033 ± 0.0023	0.8016 ± 0.0025	0.8849 ± 0.0005
DSConv(KD)	6417	0.8054 ± 0.0015	0.8031 ± 0.0018	0.8906 ± 0.0023

**Table 7:** Results of the loss component ablation experiment on the test set over three random seeds.

Configuration	Accuracy	Macro-F1	AUC-ROC
Direct (No KD, $L_{\mathrm{hard}}$ only)	0.8051 ± 0.0022	0.8038 ± 0.0017	0.8851 ± 0.0015
$L_{\mathrm{hard}}$ + $L_{\mathrm{soft}}$	0.8068 ± 0.0024	0.8051 ± 0.0022	0.8895 ± 0.0018
$L_{\mathrm{hard}}$ + $L_{aux}$	0.8094 ± 0.0019	0.8073 ± 0.0017	0.8881 ± 0.0034
Full (Proposed)	0.8087 ± 0.0031	0.8067 ± 0.0021	0.8875 ± 0.0003

**Table 8:** Results of the lead selection ablation experiment on the test set over three random seeds.

Lead	Accuracy	Macro-F1	AUC-ROC
Lead I (idx = 0)	0.7985 ± 0.0019	0.7959 ± 0.0018	0.8772 ± 0.0021
Lead II (idx = 1)	0.8087 ± 0.0031	0.8067 ± 0.0021	0.8875 ± 0.0003
Lead III (idx = 2)	0.7379 ± 0.0045	0.7362 ± 0.0058	0.8124 ± 0.0020

**Table 9:** Efficiency comparison between the teacher model and the student inference model.

Metric	Teacher	Student (GPU)	Student (Cortex-M4)
Parameters	793,733	6417	6417
FP32 size	≈3101 KB	≈25.1 KB	—
INT8 size	—	≈6.3 KB *	20.8 KB
Latency	2.46 ms (GPU)	0.38 ms (GPU)	11.6 ms (@168 MHz)
Peak SRAM usage	—	—	63.0 KB
Speedup	1×	6.4×	—
Compression	1×	123.7×	123.7×

* The 6.3 KB value refers only to the quantized model weights, whereas the 20.8 KB TFLite file includes the computational graph structure and quantization parameter tables.

**Table 10:** Manual evaluation of the base and fine-tuned models.

Evaluation Dimension	Base Qwen3-8B	Ours-FT Qwen3-8B
Medical Accuracy	3.31 ± 0.79	4.61 ± 0.61
Structural Completeness	3.89 ± 0.83	4.89 ± 0.47
End-to-End Consistency	4.14 ± 0.87	5.00 ± 0.00
Personalization	3.44 ± 0.98	4.78 ± 0.43
Safety Compliance	4.00 ± 0.84	4.61 ± 0.61
Overall Average Score	3.75 ± 0.72	4.78 ± 0.23

**Table 11:** Automated evaluation of the base model, fine-tuned model with edge-side prior, and fine-tuned model without edge-side prior.

Metric	Base	Ours-FT (w/Prior)	Ours-FT (w/o Prior)
Avg. Report Length	5081	2329	2089
Section Coverage	93.0%	92.5%	86.2%
Safety Disclaimer Rate	100.0%	98.0%	94.0%
HR Citation Rate	99.0%	100.0%	100.0%
SDNN Citation Rate	100.0%	100.0%	100.0%
BMI Output Rate	91.0%	100.0%	100.0%
BMI MAE	1.10	0.38	0.43
BMI Acc ≤ 0.5	35.0%	73.0%	73.0%

## Data Availability

The PTB-XL dataset used in this study is publicly available from PhysioNet. Additional data generated during this study are available from the corresponding author upon reasonable request.

## Abbreviations

The following abbreviations are used in this manuscript:

ECG	Electrocardiogram
KD	Knowledge distillation
LLM	Large language model
LoRA	Low-Rank Adaptation
MCU	Microcontroller unit
DSConv	Depthwise separable convolution
HR	Heart rate
HRV	Heart rate variability
SDNN	Standard deviation of normal-to-normal intervals
BLE	Bluetooth Low Energy
TFLM	TensorFlow Lite Micro
AUC-ROC	Area under the receiver operating characteristic curve
AP	Average precision
